# *Candida* Species (Volatile) Metabotyping through Advanced Comprehensive Two‐Dimensional Gas Chromatography

**DOI:** 10.3390/microorganisms8121911

**Published:** 2020-11-30

**Authors:** Carina Pedrosa Costa, Ana Rita Bezerra, Adelaide Almeida, Sílvia M. Rocha

**Affiliations:** 1 Department of Chemistry & LAQV-REQUIMTE, University of Aveiro, Campus Universitário Santiago, 3810-193 Aveiro, Portugal; carina.pedrosa@ua.pt; 2 Health Sciences Department, Institute for Biomedicine—iBiMED, University of Aveiro, Campus Universitário Santiago, 3810-193 Aveiro, Portugal; armbezerra@ua.pt; 3 Department of Biology & CESAM, University of Aveiro, Campus Universitário Santiago, 3810-193 Aveiro, Portugal

**Keywords:** *Candida* species, microbial metabolomics, solid phase microextraction, comprehensive two-dimensional gas chromatography, *Candida* spp. distinction, metabotyping

## Abstract

Microbial metabolomics is a challenge strategy that allows a comprehensive analysis of metabolites within a microorganism and may support a new approach in microbial research, including the microbial diagnosis. Thus, the aim of this research was to in-depth explore a metabolomics strategy based on the use of an advanced multidimensional gas chromatography for the comprehensive mapping of cellular metabolites of *C. albicans* and non-*C. albicans* (*C. glabrata* and *C. tropicalis*) and therefore contributing for the development of a comprehensive platform for fungal detection management and for species distinction in early growth times (6 h). The volatile fraction comprises 126 putatively identified metabolites distributed over several chemical families: acids, alcohols, aldehydes, hydrocarbons, esters, ketones, monoterpenic and sesquiterpenic compounds, norisoprenoids, phenols and sulphur compounds. These metabolites may be related with different metabolic pathways, such as amino acid metabolism and biosynthesis, fatty acids metabolism, aromatic compounds degradation, mono and sesquiterpenoid synthesis and carotenoid cleavage. These results represent an enlargement of ca. 70% of metabolites not previously reported for *C. albicans*, 91% for *C. glabrata* and 90% for *C. tropicalis*. This study represents the most detailed study about *Candida* species exometabolome, allowing a metabolomic signature of each species, which signifies an improvement towards the construction of a *Candida* metabolomics platform whose application in clinical diagnostics can be crucial to guide therapeutic interventions.

## 1. Introduction

Fungal pathogens can cause life threatening invasive infections (fungemia and meningitis), chronic conditions (asthma) and recurrent superficial infections (oral and vaginal candidiasis). Globally, the death toll has been estimated to be around 1.6 million deaths per year [1]. Species belonging to the genera *Aspergillus*, *Candida* and *Cryptococcus* are the most prevalent cause of invasive infections, and recent data indicate that *Candida* infections account for 80% of all systemic fungal infections worldwide [2]. The epidemiology of *Candida* infections is constantly changing due to changes in medical practices, but approximately 95% of all cases of *Candida* infections result from five species: *Candida albicans*, *Candida glabrata*, *Candida parapsilosis*, *Candida tropicalis* and *Candida krusei* [2]. *Candida* spp. exist as commensals of the skin, mouth and gastrointestinal tract. Their growth and spread are controlled by epithelial barriers and defenses of the innate immune system. While *Candida* spp. are normal flora of the human body, they also possess the ability to transition to pathogens causing life-threatening systemic infections [3,4,5]. Additionally, the presence of candidemia results in an increase in mortality and costs associated with prolonged hospitalization, making *Candida* spp. not only a clinical concern but also an economic concern as well [6].

*C. albicans* is present on the human microflora as a diploid yeast. One of the hallmark features of *C. albicans* is its phenotypic plasticity that promotes adaptation inside the host. This plasticity includes growing with distinct morphologies, yeast, hyphae, pseudohyphae and chlamydospores. Yeast cells have a round/oval shape, similar to *Saccharomyces cerevisiae* morphology that contrasts with the thin and tube-shaped morphology of hyphal cells [7]. Both morphological forms seem to have distinct functions during the different stages of infection, including adhesion, invasion, damage, dissemination, immune evasion and host response. The hyphal form has been shown to be more invasive while the smaller yeast form is believed to be mainly involved in dissemination [7,8]. The other main characteristic that establishes *C. albicans* as a successful pathogen is its huge adaptability, allowing it to grow on the most diverse microenvironments in the host niches (with different nutrient availability, pH, hypoxia and CO_2_ levels) [9,10]. Unlike many other commensal microbes, that require specific carbon sources in order to proliferate (e.g., glucose), *C. albicans* has the capacity of metabolizing a diversity of carbon sources present in different host niches, such as sugars, fatty acids, amino acids and short chain carboxylic acids (lactate) by differential activation of glycolysis, gluconeogenesis or the glyoxylate cycle. Besides that, *C. albicans* is also able to mount strong stress responses and can survive periods of starvation, providing it the fitness attributes that are crucial for its survival in the hostile environment of the host [10,11].

In the phylogenetic tree, *C. glabrata* and *C. albicans* are separated by several non-pathogenic yeasts, suggesting that the ability to infect humans has evolved independently [12]. Differences between the two species include the fact that *C. glabrata* is strictly haploid and only grows in the yeast form. Additionally, unlike the high metabolic flexibility of *C. albicans, C. glabrata* appears more specialized in its metabolic requirements [13]. In fact, *C. glabrata* lacks many of the metabolic pathways known in other yeasts. For example, it cannot catabolize galactose and allantoin, and is auxotrophic for pyridoxine, nicotinic acid and thiamine [12]. *C. tropicalis* exhibits several phenotypic traits associated with *C. albicans*, namely the ability to produce true hyphae and biofilms [14].

In clinical laboratories, fungal infections can be detected based on conventional methods, such as cell culture and following identification by phenotypic, immunologic and genotypic methods [15]. While culture and microscopy remain the standard techniques for diagnosis, sensitivity and specificity of such methods are limited [16,17,18]. Immunologic tests are not suitable for immunocompromised patients, for which fungal infection is a serious problem, although they allow faster results. Therefore, several commercial PCR-based detection methods are now available for a more sensitive and targeted diagnostic of fungal infections [15,19]. Additionally, other molecular methods are being developed for rapid detection of antifungal resistance [20]. The delayed diagnosis and therapy contribute to the high mortality rates, thus the development and implementation of rapid, accurate and cost-effective laboratory diagnosis is very important to provide help and selection of appropriate antifungal therapy, alongside the enhanced drug stewardship [19,21,22].

Microbial metabolomics is a comprehensive analysis of metabolites within a microorganism that may help to better understand complex biological systems and supports a new approach in microbial research, including the microbial diagnosis. The microorganisms produce a wide range of metabolic products, which are the final products of biochemical processes and a result of environmental and genetic interactions, such as volatile metabolites that can be used as unique volatile metabolic fingerprints of each species, and possibly of strains [21,23,24]. Genetic differences promote differential expressions of genes implicated in the biosynthesis of volatile metabolites, or even promote different metabolites concentrations [23,24]. Metabolomic profiling may provide additional information about the processes affected by specific microorganisms or microbiota alterations or about the environment affects the organisms that are susceptible to environmental changes and stress conditions [24,25].

**Figure 1:**
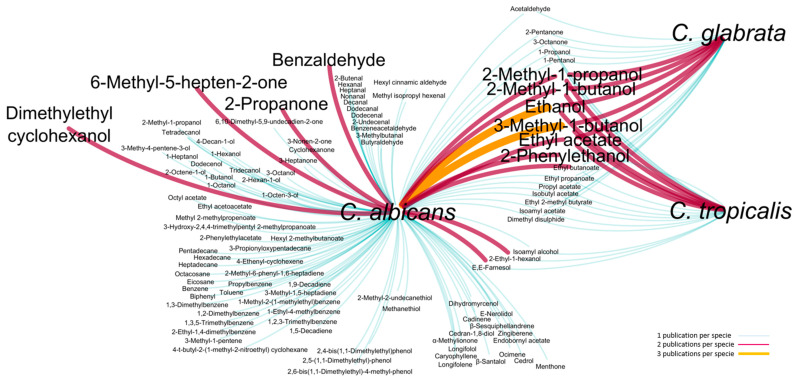
Visual representation of the volatile metabolites reported in the literature for the *C. albicans*, *C. glabrata* and *C. tropicalis*. Nodes represent *Candida* species, and the lines make the connection between species and metabolites, some of them common to 2 or 3 species. The color and line thickness represent the number of citations of each metabolite per specie: 1 (white blue), 2 (violet) and 3 (orange) publications [26,27,28,29,30,31,32,33,34,35].

Some studies are already performed regarding the profiling of volatiles metabolites from *C. albicans* and non-*C. albicans*, such as, *C. glabrata* and *C. tropicalis*. A visual representation was constructed using the data available in the literature for the *C. albicans*, *C. glabrata* and *C. tropicalis* (Figure 1): the nodes represent *Candida* species and the lines make the connection between species and metabolites, some of them common to 2 or 3 species [26,27,28,29,30,31,32,33,34,35]. This figure unveils that limited information is currently available about the metabolomics profile of these *Candida* species. The benzaldehyde, dimethylethyl cyclohexanol, ethanol, 2-methyl-1-propanol, 2-methyl-1-butanol, 3-methyl-1-butanol, 2-phenylethanol, 2-ethyl-1-hexanol, 2-propanone, 6-methyl-5-hepten-2-one, ethyl acetate, ethyl butanoate and *E,E*-farnesol are the most cited metabolites (Figure 1). The common metabolites among the three *Candida* species are ethanol, 1-propanol, 1-pentanol, 2-methyl-1-propanol, 2-methyl-1-butanol, 3-methyl-1-butanol, 2-phenylethanol, ethyl acetate, ethyl propanoate, propyl acetate, isobutyl acetate, ethyl butanoate, 2-pentanone, 3-octanone and dimethyl disulfide. Particular attention has been given to *E,E*-farnesol, an extracellular quorum sensing molecule produced by *C. albicans* [36], that represses the induction of hyphal growth by yeast cells in many different environments [37]. This metabolite was already reported for *C. tropicalis* [34].

In fact, these microbial metabolomics studies used in general one-dimensional gas chromatography (1D-GC) [26,28,30,31,32,33,34,35], however, the data from their metabolome may be enlarged if a high sensitive and high throughput technique will be used. Recent advances in metabolomics studies applied to a wide range of microbes have been used successfully using the comprehensive two-dimensional gas chromatography coupled to mass spectrometry with a time of flight analyzer (GC×GC–ToFMS) [21,23,27,38,39,40,41,42,43]. GC×GC employs two orthogonal mechanisms to separate the constituents of the sample within a single analysis, based on the application of two GC columns coated with different stationary phases. The interface samples small (several seconds) portions of the first dimension (^1^D) eluate, in general, by cryofocusing, and reinjects them into the second column (^2^D). Each ^1^D peak is modulated several times, largely preserving the ^1^D separation. Using this instrumental approach, compounds coeluting from ^1^D undergo additional separation on ^2^D [4]. Therefore, sensitivity and limits of detection (LoD) are improved due to focusing of the peak in the modulator and separation of analytes from the chemical background [44,45]. The signal-to-noise ratio was enhanced for GC×GC, compared to D-GC. Additionally, the combination of the GC×GC and a mass spectrometer, with a time-of-flight analyzer (ToFMS), allowed the detection and quantification of analytes in the range of pg. Indeed, the narrow peaks produced by GC×GC (peak width at half height of 0.1 s or less) require a detector with high data acquisition speed (ca. hundred full-mass-range spectra per second), such as ToFMS, thus providing sufficient data density. Moreover, ToFMS allows the acquisition of full mass spectra at trace levels and mass spectral continuity, letting a reliable spectra deconvolution of overlapping peaks.

Thus, the aim of this research was to in-depth explore a metabolomics strategy for the comprehensive mapping of cellular metabolites of pathogens, namely *C. albicans*, *C. glabrata* and *C. tropicalis*, therefore contributing for the development of a comprehensive platform for fungal detection management and for species distinction in early growth times (6 h). Due to its high clinical relevance, in this study particular attention was done for *C. albicans*, and for comparative purposes, other non-*C. albicans* species were also studied. The metabolomic profiles were characterized during a time-course experiment through a methodology based on headspace solid-phase microextraction (HS-SPME), a green and solvent free extraction technique, combined with GC×GC–ToFMS. The metabolomics data were subjected to hierarchical cluster analysis, allowing differentiation between the *Candida* spp. This study also allowed the identification of a large set of new compounds compared to previous studies, representing an improvement towards the construction of a *Candida* spp. omics pipeline. Finally, the set of identified metabolites was integrated, as possible, in the corresponding pathways network associated with yeasts metabolism. Although further studies are needed, our work intends to improve knowledge on the metabolic flexibility of *Candida* species as a strategy for virulence.

## 2. Materials and Methods

**Figure 2:**
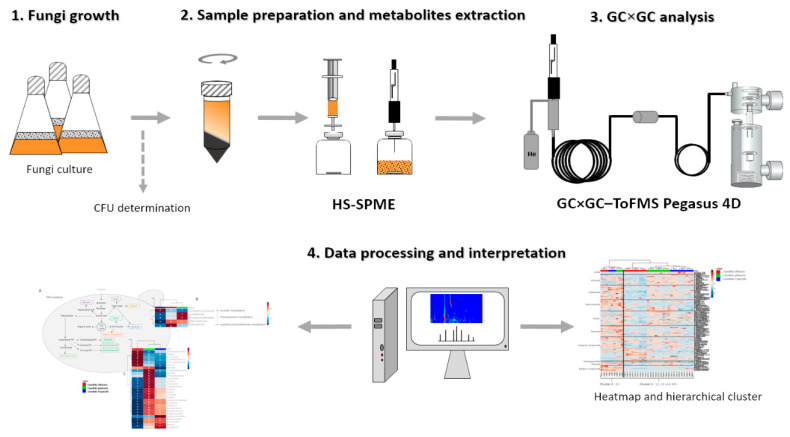
Workflow representation of the main stages for *Candida* species exometabolome analysis. The stages include yeast growth, sample preparation and metabolites extraction, GC×GC analysis, data processing and interpretation. Three independent assays were performed for each species.

The fungi growth, sample preparation and extraction, instrumental analysis and information relative to data preprocessing, pretreatment, processing and interpretation were done according to the metabolomics standards initiative (MSI) [46,47,48]. The main stages of experimental procedure were performed according to Figure 2, and include yeast growth, sample preparation, metabolites extraction, GC×GC analysis and data processing, which are described in the following subsections.

### 2.1. Yeasts Species and Growth Conditions

Three yeasts were used in this study: *Candida albicans* SC5314 (ATCC MYA-2876)*, Candida tropicalis* DSY472 (ATCC 750) and *Candida glabrata* NCCLS 84 (ATCC 90030). Fresh cultures were obtained by streaking each species on yeast glucose chloramphenicol (10 gL^−1^ d-glucose and 5 gL^−1^ yeast extract). Firstly, to evaluate the impact of growth conditions on metabolite production, *C. albicans*, *C. tropicalis* and *C. glabrata* were incubated in liquid YGC, at 37 °C, and incubation periods (6, 12, 24 and 48 h). For each assay, 1 flask was prepared suspending the cultures in 50 mL of YGC (10 gL^−1^ d-glucose and 5 gL^−1^ yeast extract). Three independents assays were done for each condition, corresponding to a total of 3 flasks per condition (i.e., 3 yeast species × 4 growth times, each one by 3 independent assays = a total of 36 flasks). From each flask, it was collected 25 mL for metabolites profiling and 25 mL for cell concentration determination. Cell concentration was determined as colony-forming units per milliliter (CFU mL^−1^). The homogenized suspension was serially diluted in Ringer solution and aliquots of 100 μL were spread on YGC_A_ (5 replicates per dilution). The cell concentration of each aliquot was used to normalize the total areas of each chemical feature detected, therefore allowing the determination of specific metabolite production per cell.

### 2.2. Exometabolome Profiling of Candida Species Cultures

After incubation, 25 mL of each sample (YGC culture broth and respective medium control) were collected and centrifuged at 10,000 rpm, at 4 °C for 15 min (Centrifuge Beckman AVANTI). For HS-SPME procedure, 20 mL (1/β ratio of 0.5) of supernatant were transferred into a 60 mL glass vial, via a syringe with a 0.20 μm filter pore. After the addition of 4 g of NaCl (≥99.5%, Sigma-Aldrich, St. Louis, MO, USA) and stirring bar of 20 mm × 5 mm, the vial was capped with a silicone/polytetrafluoroethylene septum and an aluminum cap (Chromacol Ltd., Herts, UK). The samples were stored at −80 °C until analysis.

The SPME holder for manual sampling and the coating fiber were purchased from Supelco (Aldrich, Bellefonte, PA, USA). The selected SPME device included a fused silica fiber coating, partially cross-linked with 50/30 μm divinylbenzene/carboxen™/polydimethylsiloxane StableFlex™ (1 cm), which comprehends a wide range capacity of sorbing compounds with different physicochemical properties [36]. After defrosting, the vials were placed in a thermostated water bath and the headspace extraction was allowed to occur for 30 min, at 50 °C and under continuous agitation at 350 rpm. Three independent aliquots were analyzed for each condition under study.

The SPME fiber was manually introduced into the GC×GC–ToFMS injection port and exposed during 30 s for thermal desorption into the heated injection port (250 °C). The instrumental parameters were defined according to a previous metabolomics studies [27,49]. The injection port was lined with a 0.75 mm I.D. splitless glass liner and splitless injections mode were used (30 s). The LECO Pegasus 4D (LECO, St. Joseph, MI, USA) GC×GC–ToFMS system comprised an Agilent GC 7890A gas chromatograph (Agilent Technologies, Inc., Wilmington, DE, USA), with a dual stage jet cryogenic modulator (licensed from Zoex) and a secondary oven, and mass spectrometer equipped with a ToF analyzer. An Equity-5 column (30 m × 0.32 mm I.D., 0.25 μm film thickness, Supelco, Inc., Bellefonte, PA, USA) and a DB-FFAP column (0.79 m × 0.25 mm I.D., 0.25 μm film thickness, J&W Scientific Inc., Folsom, CA, USA) were used for first (^1^D) and second (^2^D) dimensions, respectively. The carrier gas was helium at a constant flow rate of 2.50 mL min^-1^. The following temperature programs were used: the primary oven temperature ranged from 40 (1 min) to 140 °C at 10 °C min^−1^, and then to 200 °C (1 min) at 7 °C min^−1^. The secondary oven temperature program was 15 °C offset above the primary oven. Both the MS transfer line and MS source temperatures were 250 °C. The modulation period was 5 s, keeping the modulator at 20 °C offset above the primary oven, with hot and cold pulses by periods of 0.80 and 1.70 s, respectively. The ToF analyzer was operated at a spectrum storage rate of 100 spectra s^−1^, with a mass spectrometer running in the EI mode at 70 eV and detector voltage of −1499 V, using an *m/z* range of 35–350. Total ion chromatograms were processed using the automated data processing software ChromaTOF^®^ (LECO) at a signal-to-noise threshold of 200. For identification purposes, the mass spectrum and retention times (^1^D and ^2^D) of the analytes were compared with standards, when available. Additionally, the identification process was done by comparing the mass spectrum of each peak with existing ones in mass spectral libraries, which included an in-house library of standards and two commercial databases (Wiley 275 and US National Institute of Science and Technology (NIST) V. 2.0—Mainlib and Replib). Moreover, a manual analysis of mass spectra was done, combining additional information like linear retention index (RI) value, which was experimentally determined according to van Den Dool and Kratz RI equation [50]. A C_8_–C_20_ *n*-alkanes series was used for RI determination (the solvent *n*-hexane was used as the C_6_ standard), comparing these values with reported ones in existing literature for chromatographic columns similar to the ^1^D column mentioned above. The majority of the identified compounds (>90%) presented similarity matches >800/1000. The deconvoluted total ion current GC×GC area data were used as an approach to estimate the relative content of each metabolite in the samples.

### 2.3. Statistical Analysis

Firstly, the peak areas data of all metabolites were extracted from the chromatograms and used to build the full data matrix from *Candida* species cultures consisting of 36 observations (3 yeast species × 4 growth times, each one by 3 independent assays) and 129 variables. The complete list of these analytes is provided in Supplementary Information Table S1, including the areas of the three independent assays of each condition under study. The significance of the analytes detected in the yeast cultures (absolute GC areas) were compared to the ones that were detected in the YGC medium (control), through a two-sided Mann–Whitney test (using the SPSS software 20.0 (IBM, New York, NY, USA)). Differences corresponding to *p* < 0.05 were considered significant. Thus, analytes that had the same statistical value between YGC medium and samples were excluded (*p* > 0.05, analytes were marked with * in Supplementary Information Table S1), reducing the data from 129 to 126 variables (data set used for statistical analysis): of 36 observations (3 yeast species × 4 growth times, each one by 3 independent assays) and 126 variables.

A hierarchical cluster analysis (HCA) combined with the heatmap visualization was applied for this dataset, the area of each variable was autoscaled and normalized by the sum for all samples (with GC peak previously normalized by CFU mL^−1^) using the MetaboAnalyst 3.0 (web software, The Metabolomics Innovation Centre (TMIC), Edmonton, AB, Canada). Moreover, two heatmaps were constructed with metabolites from the amino acid metabolism and the terpen-secondary metabolites, each one with 9 observations (3 yeast species × 1 growth times—6 h, each one by 3 independent assays) and 5 variables (metabolites related with amino acid metabolism) and 29 variables (terpenic compounds), respectively. The significance of the analytes detected among the 3 species were compared, through a two-sided Mann–Whitney test (using the SPSS software 20.0 (IBM, New York, NY, USA)). Differences corresponding to *p* < 0.05 were considered significant.

## 3. Results and Discussion

### 3.1. Candida spp. Exometabolome

In general, the growth conditions were established according to methods currently performed in clinical laboratories. The conventional procedures leading to yeast identification are often based on yeast growth on solid media, normally for 24–72 h [51]. However, in order to reduce the analysis time, different growth times were evaluated (6, 12, 24 and 48 h), corresponding to the different cell cycle phases: 6 h corresponds to the end of lag phase; 12 h matches around the middle of the exponential phase; 24 h coincides with the end of the exponential phase and beginning of the stationary phase; and at 48 h yeasts are in the stationary phase [52,53]. Thus, to determine the volatile headspace components of *Candida* species cultures, samples were grown at 37 °C during 6–48 h, and metabolites were capture from headspace using SPME, as described in the material and methods section (2.2. Exometabolome profiling of Candida species cultures). After GC×GC instrumental analysis, data were collected for matrices construction with GC×GC peak area data and for metabolites identification.

**Figure 3:**
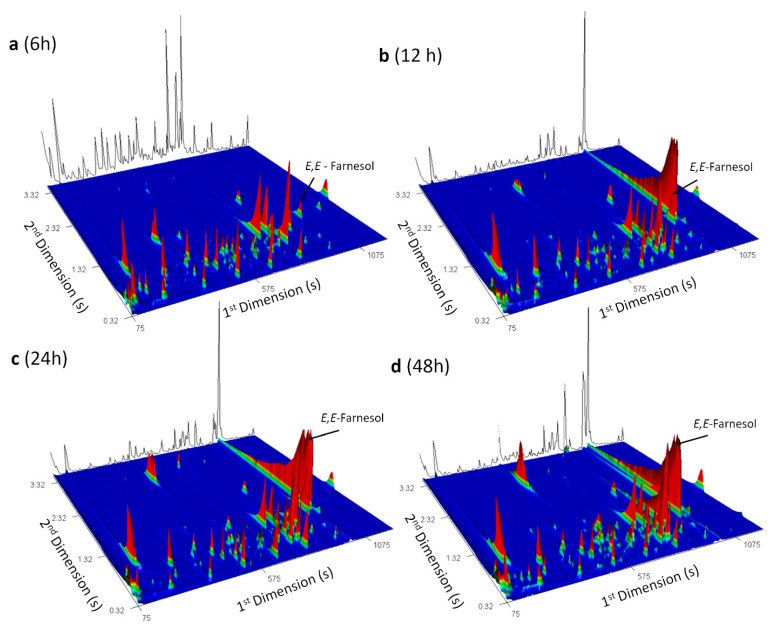
Selected GC×GC–ToFMS total ion chromatogram contour plot of the *C. albicans* cultures headspace volatile compounds at four growth times: (**a**) 6 h, (**b**) 12 h, (**c**) 24 h and (**d**) 48 h of culture headspace volatile compounds, used to illustrate a yeast metabolomic profile. *E,E-*Farnesol, an important quorum-sensing molecule, was highlighted.

For instance, Figure 3 shows the total ion chromatogram contour plot of the *C. albicans* cultures headspace volatile compounds at the four growth times: (a) 6 h, (b) 12 h, (c) 24 h and (d) 48 h. These contour plots may be used as pictures of the *C. albicans* metabolome over time and unveil the data complexity*. E,E*-Farnesol were highlighted to reveal differences over time of growth, and it can be pointed out that the content of this metabolite increased until 24 h and then a decrease was observed for 48 h (Supplementary Information Figure S1). For *C. glabrata* and *C. tropicalis*, *E,E*-farnesol is not detected for the shortest growth time (6 h), which represent a particular characteristic of *C. albicans*. 

Typically, the GC×GC contour plots from *Candida* species contain ca. 750 instrumental features (Figure 3), which identification represents a huge challenge. Thus, a comprehensive strategy was performed, based on the combination of the coinjection of standards, when available, and the analysis of acquired mass spectrum, RI and the comparison of these two chromatographic data with homemade and commercial databases. Additionally, the GC×GC structured chromatogram principle, a particular characteristic of the bidimensional GC, was used as a helpful tool. GC×GC structured chromatogram principle was a powerful tool in the identification procedure since compounds structurally related should be on similar 2D chromatographic space. Considering the set of columns used (non-polar/polar), the decrease in volatility (high ^1^*t*_R_) is mainly related to the increasing in the number of carbons through the ^1^D. Otherwise, increasing in the ^2^*t*_R_ corresponds to polarity increasing.

**Figure 4:**
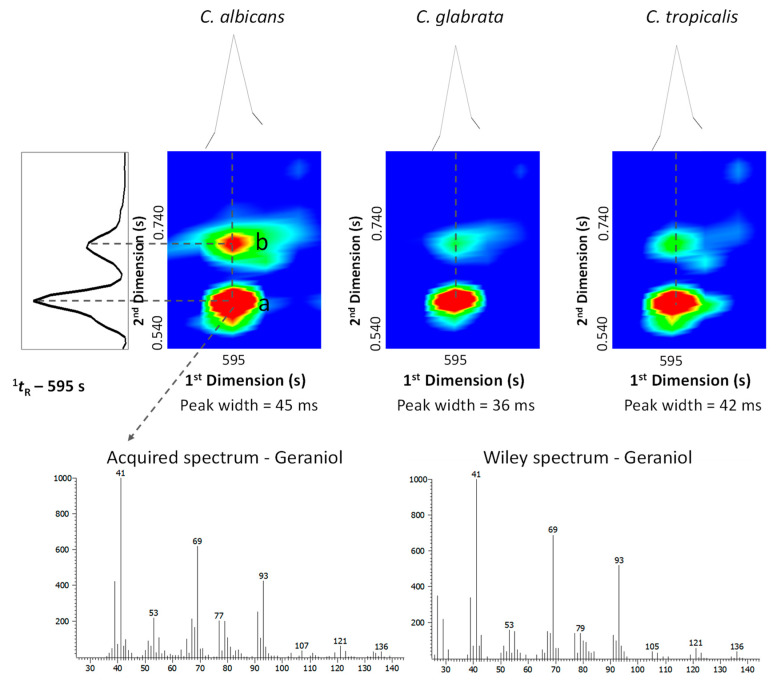
Blow-up of a part of GC×GC chromatogram contour plots from the *C. albicans*, *C. glabrata* and *C. tropicalis* (12 h) showing the separation, through the ^2^D, of (a) geraniol (C_10_H_18_O) and (b) 2-isoamyl-6-methyl pyrazine (C_10_H_16_N_2_), metabolites that exhibited the same retention time in the ^1^D. The 36–45 milliseconds wide geraniol GC×GC peak was identified at a mass spectral acquisition of 100 spectra/s. The geraniol acquired mass spectrum presented a similarity value of 939/1000 compared to the Wiley database.

Figure 4 exhibits a blow-up of a part of three contour plots from the *Candida* species under study and illustrates the advantages of GC×GC–ToFMS, which allows the separation through the secondary ^2^D column of analytes with similar volatility. For instance, geraniol (C_10_H_18_O) and 2-isoamyl-6-methyl pyrazine (C_10_H_16_N_2_) present similar volatility, and consequently they exhibited the same ^1^*t*_R_ (595 s). However, they were separated by the ^2^D column (^2^*t*_R_—0.610 and 0.710 s, respectively) as they present different polarities. The compound 2-isoamyl-6-methyl pyrazine exhibited a LogP value slightly smaller than the geraniol (LogP—2.37 and 2.67, respectively, data obtained from https://www.chemeo.com/), which supports the relatively lower polarity of the geraniol and therefore the lower retention time in the ^2^D. Another important feature of the GC×GC–ToFMS is related to its sensitivity and spectral quality of the acquired data, which are crucial for identification purposes. A practical example can be observed in Figure 4 for geraniol, a trace component, presenting a 36–45 milliseconds wide GC×GC peak, which was identified at a mass spectral acquisition of 100 spectra/s. Spectral quality at this high acquisition rate is maintained due to the ToFMS with a continuous full-range mass spectral acquisition rate. As observed, the geraniol acquired mass spectrum was very similar (similarity value of 939/1000) compared to the Wiley database.

The complete list of putatively identified metabolites is available in Table 1 and Supplementary Information Table S1. The GC×GC–ToFMS, a high sensitive and high throughput methodology, revealed the complexity of the matrices under study, as reflected in the dataset produced. From, all the detected instrumental features, a total number of 129 metabolites were putatively identified in *Candida* species metabolome, which are distributed over several chemical families such as acids, alcohols, aldehydes, hydrocarbons, esters, ketones, terpenic compounds including monoterpenic compounds and sesquiterpenes, norisoprenoids, phenols and sulphur compounds. This set of metabolites were reducing from 129 to 126, after removing the analytes that did not exhibit statistically significant differences (*p* > 0.05) between samples and YGC medium composition, used as background control: 3-methylheptyl acetate, levomenthol and α-terpineol (Supplementary Information Table S1).

**Figure 5:**
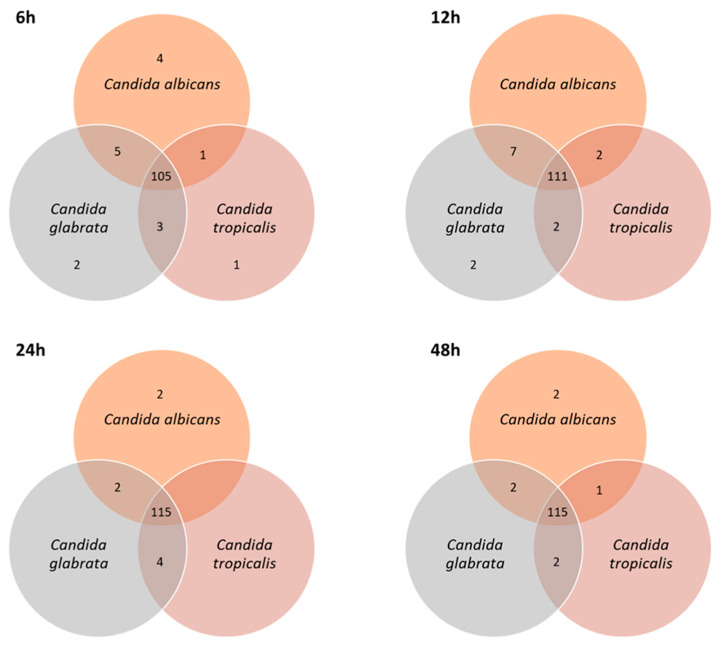
Total number of shared and unique metabolites of each *Candida* species over the time-course experiment.

Considering this data set of 126 compounds, the number of metabolites per sample varied between 111 for *C. tropicalis* (6 h) to 123 for *C. glabrata* (12 h; Supplementary Information Tables S2 and S3). Apart from acids that were not detected at 6 h for any of the species under study, there were components of the different chemical families in the four sampling moments. The number of shared and unique metabolites of each *Candida* species changed over the time-course experiment (6–48 h of growth; Figure 5). The number of shared compounds by all species was slightly higher (9.5%) for longer growth times, which ranged from 105 at 6 h to 115 compounds at 24 h and 48 h. At 6 h of growth time, α-farnesene isomer, 2,3-dihydrofarnesol, *E,E*-farnesol and farnesal were the four unique metabolites of *C. albicans*, which may be further included in a potential pattern of biomarkers for the early contamination stage.

Only 30% of the identified metabolites were previously reported in the literature for *C. albicans* samples, 9% for *C. glabrata* and 10% for *C. tropicalis*, as shown in Table 1. This represents a clear improvement from previous studies and validates this methodology for future studies of *Candida* spp. metabolism. The high number of metabolites that characterize the headspace of *Candida* species cultures allowed us to understand a network of pathways that may explain the origin of several detected metabolites (Table 1). 

Altogether, this study allowed the identification of more compounds compared to previous studies, but more importantly, it allowed the detection of new metabolites that were never reported for these *Candida* species. These metabolites can be explored successfully to distinguish *C. albicans* from other *Candida* species with 6 h of growth time, which allows to infer that this data open opportunities in the future, it might be possible to distinguish *Candida* species at an early time and doing an early diagnostic of a microbiological agent can be possible and initiating the specific treatment, because often initiating empirical therapies based on clinical evaluation of patients, without having specific information on the etiological agent, impairs their treatment [54]. Additionally, the development and implementation of faster, accurate and cost-effective detection tests are extremely important in clinical studies.

### 3.2. Exploring the Potential of Exometabolome on Candida Species Distinction

**Figure 6:**
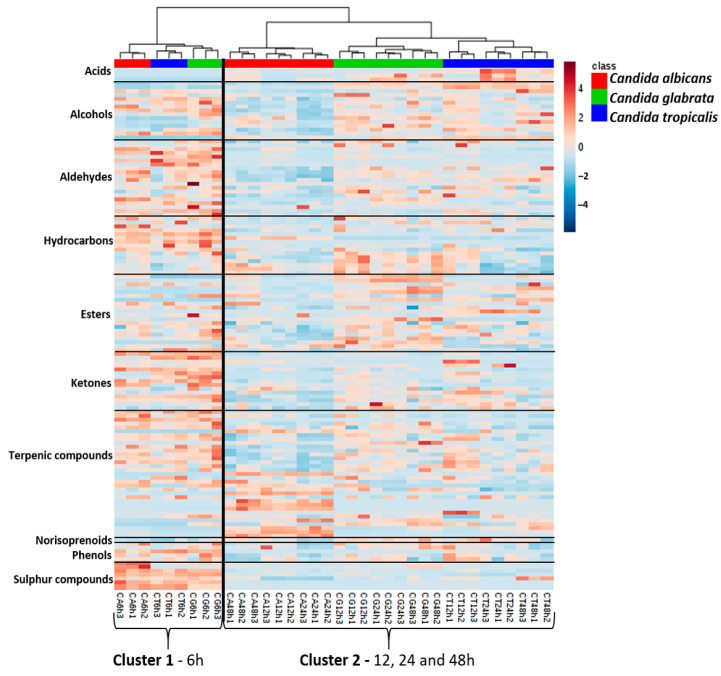
Heatmap and hierarchical cluster analysis representation of 126 metabolites putatively identified from *Candida* species cultures. The heatmap comprises the data obtained from the cultures of *C. albicans*, *C. glabrata* and *C. tropicalis* at 37 °C and 6, 12, 24 and 48 h of growth time. Cluster 1—samples from 6 h of growth, and Cluster 2—samples from 12, 24 and 48 h of growth. The area of each variable was autoscaled and normalized by the sum for all samples (with a GC peak previously normalized by CFU mL^−1^). *n* = 3 for each condition under study. The content of each metabolite was illustrated through a chromatic scale (from blue, minimum, to red, maximum). Hierarchical cluster analysis using the Ward’s cluster algorithm to the data set was also included.

A hierarchical cluster analysis (HCA) combined with the heatmap representation was constructed for an easy, rapid and global assessment of the *Candida* species metabolome over growth time and to evaluate the similarities and or differences between *Candida* species (Figure 6). The content of each metabolite was illustrated through a chromatic scale (from blue, minimum, to red, maximum), allowing a visual assessment of the relative abundance of each putatively identified compound.

It was possible to observe the formation of two main clusters: cluster 1—samples from 6 h of growth, and cluster 2—samples from 12, 24 and 48 h of growth, both including *C. albicans*, *C. glabrata* and *C. tropicalis*. In cluster 2 was possible to observe the formation of two secondary clusters: one of the *C. albicans* samples and other of *C. glabrata* and *C. tropicalis*, which allows one to infer a higher similarity between these two former species, taken into account the data set under study. The clustering analysis also reveals that although growth time is a fundamental factor for the metabolomic profile, for short or longer growth times, it is observed a clear distinction between the 3 species (Figure 6).

In fact, *C. albicans*, *C. glabrata* and *C. tropicalis* showed different volatile patterns for 6–48 h growth time. Higher relative content of metabolites was observed at 6 h of growth for the families of aldehydes, hydrocarbons, ketones, terpenic compounds, phenols and sulphur compounds. The *C. albicans* achieved the highest content for sulphur compounds. For 12, 24 and 48 h of growth, it was observed a higher relative abundance of acids in *C. tropicalis*, a higher relative abundance of terpenic compounds in *C. albicans*, whereas higher relative abundances of hydrocarbons and esters were detected in *C. glabrata*. While volatile sulphur compounds and phenols show a higher relative content for early growth time in the three *Candida* species, acids show an opposite trend, presenting higher content at longer times of growth. In this case, *Candida tropicalis* exhibited a higher value of acid content.

The higher relative abundance observed for 6 h of growth corresponded to the end of the lag phase, and the metabolites content tended to decrease along with growth time, except for terpenic compounds. The analysis of the heatmap allowed us to conclude that the metabolomics footprinting seems to be dependent of the species under study. The heatmap shows the high complexity of *Candida* species cultures, mainly composed of their metabolome components, and their individual relative abundance may be explained by different constraints affecting the metabolic pathways of *Candida* species, upon different growth times.

*E,E*-Farnesol, α-farnesene and farnesal showed a high relative content at 12, 24, and 48 h of growth. These results were in accordance with those reported by Martins et al. [30] showing that these metabolites are produced continuously by cells. For instance, farnesol is a quorum-sensing molecule, which is produced continuously in response to an increasing density of cells, such as the case of 24 h of continuous growth (Supplementary Information Figure S1), which are in accordance with a previous study [36]. The *E,E*-farnesol is a quorum-sensing molecule produced by *C. albicans*, namely for 6 h of growth, which has many effects, including filament inhibition of this polymorphic yeast. The response to the quorum-sensing compounds farnesol may also be mediated by the cAMP pathway, as repressive effects of these compounds on hyphal formation [36,72], a very relevant factor as the hyphal form seems to be more invasive.

**Figure 7:**
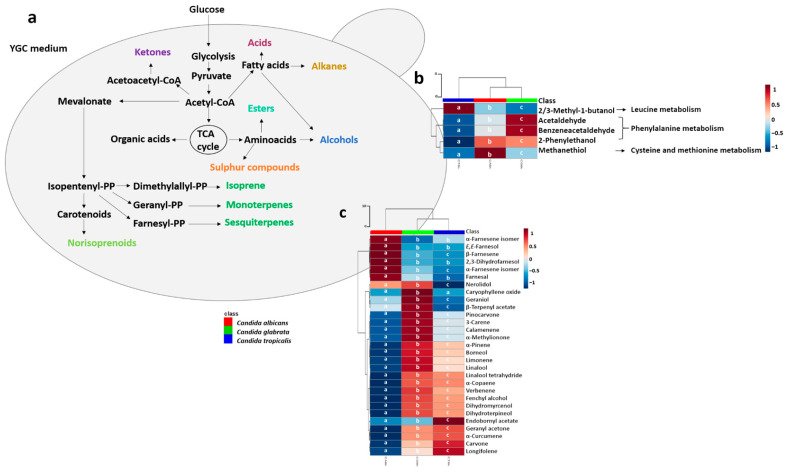
(**a**) Schematic representation proposed to explain *Candida* species metabolic pathways related to metabolites chemical families [70,71] and heatmap visualization of the metabolites from three strains of *Candida* species cultures: *Candida albicans*, *Candida glabrata* and *Candida tropicalis* at 6 h of growth time. (**b**) Amino acid metabolism related analytes; (**c**) Terpen-secondary metabolites [75]; Differences corresponding to *p* < 0.05 were considered significant and were marked with a, b and c.

Figure 7a shows the simplified schematic representation proposed to explain the metabolic pathways related to headspace released compounds from *Candida* species.

Some of the metabolites are known to play important roles in the biosynthesis of unsaturated fatty acids, such as ketones. 2/3-methyl-1-butanol and 2-phenylethanol were produced by the degradation of isoleucine/leucine and phenylalanine, respectively. 2-methyl-1-propanol was produced by the degradation of valine. Aldehydes and alcohols have also been related to fatty acids metabolism [70,73,74,75]. Esters can be produced through an enzyme-catalyzed condensation between an alcohol and an acetyl-CoA group, for example, isoamyl acetate can be produced through a reaction between 3-methyl-1-butanol and an acetyl-CoA group [76]. Sesquiterpenic compounds, such as farnesol can be biosynthesized through the mevalonate pathway, the isoprenoid precursors can be formed from acetyl-CoA, important to the formation farnesyl phosphate, that consequently promote the formation of sesquiterpenic compounds, consisting of three isoprene units [26,70]. 

Two subsets of metabolites from 6 h of growth were selected and the corresponding heatmaps were constructed that also confirm the distinction among the tree *Candida* species: amino acid related metabolites (5 metabolites) and terpenic compounds secondary metabolites (29 metabolites), respectively in Figure 7b,c. Most of those metabolites (88%) exhibited differences that were statistically significant between *Candida* species (differences corresponding to *p* < 0.05).

Figure 7b shows that *C. albicans* exhibited the highest content of methanethiol related to cysteine and methionine metabolism. Previous study [77], showed the relationship between the methionine and cysteine biosynthesis pathway and biofilm formation. The search of this pathway in the *C. albicans* genome database reveals that ECM17 encodes a putative enzyme that functions in sulphur amino acid biosynthesis. Additionally, the *C. glabrata* when comparing displays the lower biofilm metabolic activity in comparison with the other *Candida* species [78]. The higher content of methanethiol in *C. albicans* allows one to infer higher cysteine and methionine-related metabolic activity. Apart from *E,E*-farnesol, α-farnesene, β-farnesene, 2,3-dihydrofarnesol and farnesal that are detected only from *C. albicans* cultures, in general, the *C. glabrata* exhibited the highest content of terpen-secondary metabolites (Figure 7c).

With results from this study, it is already possible to distinguish *Candida albicans*, based on a wide data set of metabolites or even on targeted chemical families, as for instance amino acid related metabolites and terpen-secondary metabolites. In fact, the *Candida* species have a specific pattern of metabolites related with several pathways, which enlarge the metabolomics knowledge available about these species.

## 4. Conclusions

The implemented methodology based on HS-SPME/GC×GC–ToFMS was shown to be suitable for the profiling of *Candida* species exometabolome, which represent the crucial step in the construction of a metabolomics workflow. *Candida* species metabolome presented a wide number of chemical features showing therefore the complexity of the *Candida* matrix, in which 126 metabolites were putatively identified within and distributed over several chemical families (acids, alcohols, aldehydes, hydrocarbons, esters, ketones, terpenic compounds including monoterpenic compounds and sesquiterpenes, norisoprenoids, phenols and sulphur compounds). This research represents the most detailed study on the volatile composition of *Candida* species.

A metabolomic signature of each *Candida* species under study was established, which were used for species distinction. Therefore, based on the developed metabolomic workflow described herein, the species metabotyping were performed and further research in a broader set of *C. albicans* collected from different conditions (clinical and environmental samples and cocultures, among others) might be valuable to study the *C. albicans* biodiversity and to contribute for microbial platform construction, useful for a more global fungal management. Finally, it is important to point out that, microbial metabolomics represents an important step for microorganism’s insight and this research work represents a relevant contribution.

## Figures and Tables

**Table 1:** Metabolites putatively identified in *Candida* species using HS-SPME/GC×GC–ToFMS. The information about ID Kegg, ID YMDB and respective pathways and the chromatographic data that helps their identification are also listed.

Metabolite	ID Kegg	ID YMDB	Pathways ^a^	MSI Level ^b^	RI_Calc_^c^	RI_Lit_^d^	Previously Reported for *C. albicans, C. glabrata* and *C. tropicalis*		
**ACIDS**									
Octanoic acid	C06423	YMDB00676	Fatty acid biosynthesis	1	1192	1188 [27]	-	-	-
Nonanoic acid	C01601	YMDB01761	Biotin metabolism	1	1303	1280 [27]	-	-	-
Decanoic acid	C01571	YMDB00677	Fatty acid biosynthesis	2	1391	1379 [27]	-	-	-
**ALCOHOLS**									
**Aliphatic**									
1-Propanol	C05979	YMDB00441	Glycerolipid metabolism	1	580	580 [27]	[28]	[28]	[28]
2-Methyl-1-propanol	C14710	YMDB00573	-	1	612	612 [27]	[28,31]	[28,31]	[28,31]
1-Butanol	C06142	YMDB01386	Butanoate metabolism	1	659	644 [27]	[27]	-	-
2/3-Methyl-1-butanol	-	YMDB00567	Leucine degradation	1	718/708	718 [27]	[27,28,30,31]	[28,31]	[28,31]
1-Hexanol	-	YMDB01473	-	1	883	878 [27]	[27]	-	-
1-Heptanol	-	YMDB01475	-	1	980	975 [27]	[27]	-	-
1-Octen-3-ol	C14272	YMDB01352	-	1	990	980 [27]	[27]	-	-
2-Ethyl-1-hexanol	C02498	YMDB01330	-	2	1029	1029 [27]	[27,33]	-	[35]
1-Octanol	C00756	YMDB00808	-	1	1084	1079 [27]	[27]	-	-
1-Nonanol	C14696	-	-	2	1173	1173 [27]	-	-	-
1-Decanol	C01633	YMDB00826	-	1	1283	1278 [27]	-	-	-
2-Undecanol	-	-	-	1	1307	1301 [27]	-	-	-
1-Dodecanol	C02277	-	-	1	1476	1476 [27]	-	-	-
**Aromatic**									
2-Phenyl-2-propanol	-	-	-	2	1091	1090 [27]	-	-	-
2-Phenylethanol	C05853	YMDB01072	Phenylalanine metabolism	1	1126	1120 [27]	[27,30]	[32]	[32,34]
**ALDEHYDES**									
**Aliphatic**									
Acetaldehyde	C00084	YMDB00022	Phenylalanine metabolism	1	548	548 [27]	[29]	[28]	-
2-Propenal	-	YMDB00812	-	2	582	563 [39]	-	-	-
2-Methyl-propanal	-	-	-	2	591	576 [39]	-	-	-
3-Methylbutanal	C07329	YMDB00499	-	2	624	633 [27]	-	-	-
2-Butenal	-	-	-	2	633	633 [27]	[29]	-	-
2-Methyl-butanal	-	-	-	2	649	643 [27]	-	-	-
3-Methyl-2-butenal	-	-	-	2	792	789 [39]	-	-	-
Hexanal	-	YMDB01759	-	1	801	801 [27]	[27]	-	-
Octanal	-	YMDB00824	-	1	1001	1001 [27]	-	-	-
Nonanal	-	-	-	1	1106	1106 [27]	[27]	-	-
Decanal	C12307	YMDB01340	-	1	1212	1207 [27]	[27]	-	-
2-Undecenal	-	-	-	2	1370	1364 [27]	[27]	-	-
Dodecanal	C02278	-	-	1	1432	1407 [27]	[27]	-	-
**Aromatic**									
Benzaldehyde	C00261	YMDB01326	Toluene and xylene degradation	1	965	965 [27]	[27,33]	-	-
Benzeneacetaldehyde	-	YMDB00116	Phenylalanine metabolism	1	1046	1046 [27]	[27]	-	-
2,5-Dimethylbenzaldehyde	-	-	-	2	1220	1208 [55]	-	-	-
4-(1-Methylethyl)-benzaldehyde	-	-	-	2	1226	1243 [27]	-	-	-
α-Ethylidenbenzeneacetaldehyde	-	-	-	2	1283	1280 [39]	-	-	-
2,4,6-Trimethylbenzaldehyde	-	-	-	2	1314	1323 [56]	-	-	-
3,5-di-tert-Butyl-4-hydroxybenzaldehyde	-	-	-	2	1777	1767 [39]	-	-	-
**HYDROCARBONS**									
**Alkanes**									
Nonane	-	-	-	1	900	900 [27]	-	-	-
1-Dodecene	-	-	-	2	1195	1191 [57]	-	-	-
Heptadecane	C01816	-	-	1	1701	1701 [27]	[27]	-	-
**Aromatic**									
Toluene	C01455	-	Toluene and xylene degradation	1	753	759 [27]	[27]	-	-
Ethylbenzene	C07111	-	Ethylbenzene degradation	2	865	860 [27]	-	-	-
Styrene	C07083	-	Styrene degradation	2	889	895 [39]	-	-	-
Isopropylbenzene	C14396	-	-	2	922	927 [27]	-	-	-
Propylbenzene	-	-	-	2	953	953 [27]	[27]	-	-
α-Methylstyrene	-	-	-	2	980	985 [27]	-	-	-
1,2,4,5-Tetramethylbenzene	C14534	-	-	2	1123	1123 [27]	-	-	-
1,4-Di-tert-butylbenzene	-	-	-	2	1257	1264 [58]	-	-	-
Biphenyl	C06588	-	Biphenyl degradation	2	1389	1383 [27]	[27]	-	-
1-Butylheptylbenzene	-	-	-	2	1636	1633 [59]	-	-	-
1-Propyloctylbenzene	-	-	-	2	1648	1645 [59]	-	-	-
1-Butyloctylbenzene	-	-	-	2	1738	1731 [59]	-	-	-
1-Propylnonylbenzene	-	-	-	2	1751	1741 [59]	-	-	-
1-Ethyldecylbenzene	-	-	-	2	1776	1764 [59]	-	-	-
**ESTERS**									
**Aliphatic**									
Ethyl acetate	C00849	YMDB00569	-	1	601	601 [27]	[28,31]	[28,31]	[28,31]
Ethyl propanoate	-	YMDB01331	-	1	685	688 [39]	[28]	[28]	[28]
Isobutyl acetate	-	YMDB00572	-	2	763	769 [27]	[31]	[31]	[31]
Ethyl butanoate	-	YMDB01385	-	1	806	806 [27]	[28,33]	[28]	[28]
Butyl ethanoate	-	-	-	2	818	818 [27]	-	-	-
Isoamyl ethanoate	-	YMDB00571	-	2	877	877 [27]	-	-	-
Hexyl ethanoate	-	YMDB01384	-	2	1017	1014 [39]	-	-	-
Ethyl octanoate	C12292	YMDB01354	-	1	1195	1195 [27]	-	-	-
Ethyl nonanoate	-	YMDB01354	-	1	1301	1295 [27]	-	-	-
3-Hydroxy-2,4,4-trimethylpentyl 2-methypropanoate	-	-	-	2	1382	1376 [27]	-	-	-
Propanoic acid, 2-methyl-, 1-(1,1-dimethylethyl)- -2-methyl-1,3-propanediyl ester	-	-	-	2	1601	1607 [60]	-	-	-
Lauryl acetate	-	-	-	2	1613	1608 [38]	-	-	-
Isopropyl myristate	D02296	-	-	2	1830	1834 [60]	-	-	-
**Aromatic**									
Methyl benzoate	-	-	-	2	1101	1101 [27]	-	-	-
2-Benzylacrylic acid methyl ester	-	-	-	2	1345	1339 [27]	-	-	-
2-Octyl benzoate	-	-	-	2	1714	1708 [39]	-	-	-
**KETONES**									
2-Propanone	C00207	YMDB01701	Propanoate metabolism	1	559	559 [27]	[27,29]	-	-
2-Butanone	C02845	-	-	1	577	590 [27]	-	-	-
2-Pentanone	C01949	-	-	2	677	664 [27]	[31]	[31]	[31]
2,3-Pentanedione	-	YMDB01434	-	2	677	665 [27]	-	-	-
4-Methyl-2-pentanone	-	-	-	2	725	729 [61]	-	-	-
3-Penten-2-one	-	-	-	2	731	717 [27]	-	-	-
2,3-Heptanedione	-	-	-	2	836	836 [39]	-	-	-
5-Methyl-2-hexanone	-	-	-	2	860	860 [27]	-	-	-
4-Heptanone	-	-	-	2	871	871 [27]	-	-	-
6-Methyl-2-heptanone	-	-	-	2	953	932 [39]	-	-	-
2-Nonanone	-	YMDB01383	-	2	1095	1095 [27]	-	-	-
Phenylacetone	C15512	-	-	2	1135	1135 [27]	-	-	-
1-Phenyl-1-butanone	-	-	-	2	1258	1254 [27]	-	-	-
2-Undecanone	C01875	YMDB01592	-	2	1301	1295 [27]	-	-	-
2-Tridecanone	-	-	-	2	1501	1495 [27]	-	-	-
**TERPENIC COMPOUNDS**									
**Monoterpenic compounds**									
α-Pinene	-	-	-	1	932	937 [27]	-	-	-
Verbenene	-	-	-	2	953	958 [27]	-	-	-
3-Carene	-	-	-	1	1011	1009 [39]	-	-	-
Limonene	C06078	YMDB01727	-	1	1028	1028 [27]	-	-	-
β-Ocimene	-	-	-	2	1040	1039 [62]	-	-	-
Dihydromyrcenol	-	-	-	2	1073	1073 [27]	[27]	-	-
Linalool tetrahydride	-	-	-	2	1101	1101 [27]	-	-	-
Linalool	-	-	-	1	1101	1107 [27]	-	-	-
Fenchyl alcohol	C02344	-	Biosynthesis of secondary metabolites	2	1118	1123 [27]	-	-	-
Dihydroterpineol	-	-	-	2	1145	1142 [63]	-	-	-
Pinocarvone	C09884	-	Limonene and pinene degradation	2	1168	1168 [27]	-	-	-
Borneol	C01411	-	-	1	1173	1174 [27]	-	-	-
Carvone	C11383	YMDB01648	Limonene and pinene degradation	2	1251	1249 [39]	-	-	-
Geraniol	C01500	YMDB01700	Biosynthesis of secondary metabolites	1	1260	1265 [39]	-	-	-
Endobornyl acetate	-	-	-	2	1295	1289 [27]	[27]	-	-
β-Terpenyl acetate	-	-	-	2	1357	1351 [27]	-	-	-
**Sesquiterpenic compounds**									
α-Copaene	-	-	-	2	1357	1370 [64]	-	-	-
Longifolene	C09699	-	Biosynthesis of secondary metabolites	2	1418	1413 [27]	[33]	-	-
β-Farnesene	-	-	-	1	1460	1458 [39]	-	-	-
Geranyl acetone	-	YMDB01701	Propanoate metabolism	2	1460	1458 [39]	-	-	-
α-Curcumene	-	-	-	2	1494	1486 [39]	-	-	-
α-Farnesene isomer	-	-	-	2	1501	1495 [65]	-	-	-
α-Farnesene isomer	-	-	-	2	1519	1515 [39]	-	-	-
Calamenene	-	-	-	2	1530	1530 [27]	-	-	-
Nerolidol	C09704	-	Biosynthesis of secondary metabolites	1	1566	1566 [27]	[30]	-	-
Caryophyllene oxide	C16908	-	-	2	1612	1610 [66]	-	-	-
2,3-Dihydrofarnesol	-	-	-	2	1695	1696 [67]	-	-	-
*E,E*-Farnesol	C01493	YMDB00404	Biosynthesis of secondary metabolites	1	1731	1730 [39]	[26,30]	-	[34]
Farnesal	C03461	-	Sesquiterpenoid biosynthesis	2	1751	1744 [39]	-	-	-
**NORISOPRENOID**									
α-Methylionone	-	-	-	2	1489	1482 [27]	[27]	-	-
**PHENOLS**									
2-(1,1-Dimethylethyl)-4-methylphenol	-	-	-	2	1366	1360 [27]	-	-	-
5-Methyl-2,4-diisopropylphenol	-	-	-	2	1466	1496 [68]	-	-	-
2,4-Bis(1,1′-dimethylethyl)phenol	-	-	-	2	1520	1514 [27]	[27]	-	-
Nonylphenol	C14993	-	-	2	1760	1720 [69]	-	-	-
**SULPHUR COMPOUNDS**									
Methanethiol	C00409	YMDB00062	Cysteine and methionine metabolism	2	563	551 [39]	[29]	-	-
Dimethyl disulfide	C08371	YMDB01438	-	2	725	728 [27]	[27]	[31]	[31]
Thiazole	-	-	-	2	736	727 [39]	-	-	-
2-Methyl-thiophene	-	-	-	2	763	759 [27]	-	-	-
3-(Methylthio)propanal	-	YMDB01466	-	2	912	912 [27]	-	-	-
Dimethyl trisulfide	C08372	YMDB01438	-	2	969	969 [27]	-	-	-

^a^ Metabolic pathways were also confirmed in Mbrole 2.0 related with Kegg [70] and Yeast Metabolome Data Base (YMDB) [71], being their KEGG and YMDB number mentioned; ^b^ Level of metabolite identification according to [48]. (1) Identified compounds; (2) Putatively annotated compounds; (3) Putatively characterized compound classes; (4) Unknown compounds; ^c^ RI_calc_: linear retention index obtained through the modulated chromatogram; ^d^ RI_lit_: linear retention index reported in the literature for one dimensional GC or GC×GC with a 5%-Phenyl-methylpolysiloxane GC column or equivalent.

## Supplementary Materials

The following are available online at https://www.mdpi.com/2076-2607/8/12/1911/s1. Table S1. Full data set used for statistical analysis including the volatile components identified by HS-SPME/GC×GC-ToFMS from the headspace of C. albicans, C. glabrata and C. tropicalis strains cultures, along growth time: list of metabolites, respective information related with ID Kegg, ID YMDB, pathways and chromatographic area normalized by CFU mL−1. Table S2. Number of the metabolites within each chemical family, considering the growth times of Candida species and number of metabolites detected. Table S3. Metabolites detected for each Candida species over the time-course experiment. Figure S1. Cromatographic area and colony-forming units (CFU) mL−1 determined during a time-course 24 experiment for C. albicans (a), C. tropicalis (b) and C. glabrata (c).
