# Supplementary material for: Candida Species (Volatile) Metabotyping through Advanced Comprehensive Two‐Dimensional Gas Chromatography
Source: Microorganisms. 2020 Nov 30;8(12):1911. doi: 10.3390/microorganisms8121911 (PMC7760324; doi:10.3390/microorganisms8121911)
Supplement: Supplementary file 1 [file microorganisms-08-01911-s001.zip › Supplementary Information - Figure S1 and Tables S2 and S3_Revised.pdf]

Candida species (volatile)metabotyping through advanced comprehensive two-dimensional gas chromatography

Carina Pedrosa Costa[1], Ana Rita Bezerra[2], Adelaide Almeida[3*], Sílvia M. Rocha[1*]
[1] Department of Chemistry & LAQV/REQUIMTE, University of Aveiro, Aveiro, Portugal; carina.pedrosa@ua.pt (C.P.C.); smrocha@ua.pt (S.M.R.)
[2] Department of Medical Sciences and Institute of Biomedicine - iBiMED, University of Aveiro, Aveiro, Portugal; armbezerra@ua.pt (A.R.B.)
[3] Department of Biology & CESAM, University of Aveiro, Aveiro, Portugal; aalmeida@ua.pt (A.A.)

* Correspondence: smrocha@ua.pt (S.M.R.); Tel: + 351 234401524; aalmeida@ua.pt (A.A.); Tel : + 351 234 370784 ;

Supplementary Information

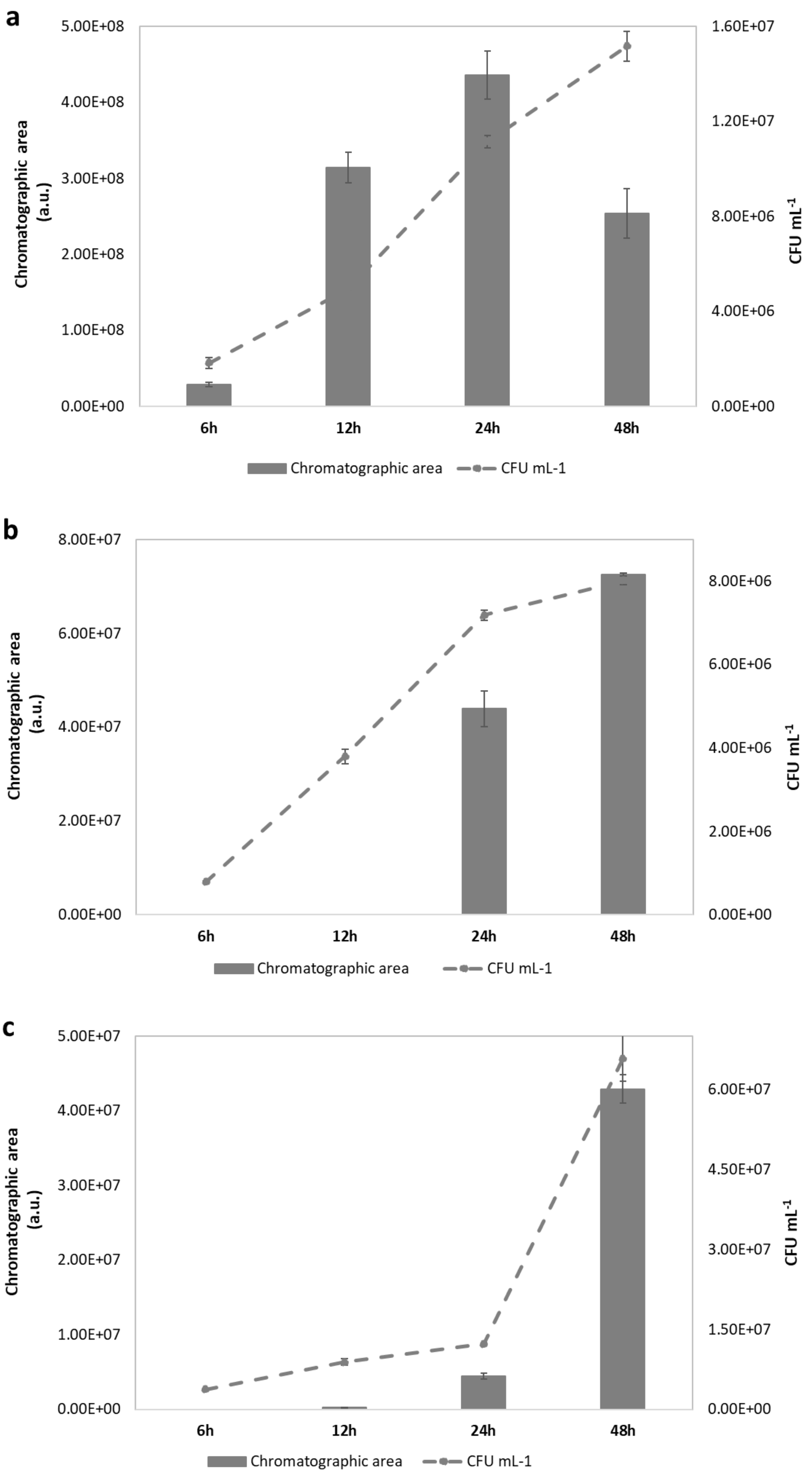

Figure S1 – Cromatographic area and colony-forming units (CFU) mL^{-1} determined during a time-course experiment for *C. albicans* (a), *C. tropicalis* (b) and *C. glabrata* (c). a. u. arbitrary units.

Table S2 – Number of the metabolites within each chemical family, considering the growth times of *Candida* species and number of metabolites detected.

Chemical family	Number of compounds	Growth times/number of metabolites detected											
		6h			12h			24h			48h		
		C. albicans	*C. glabrata*	*C. tropicalis*	*C. albicans*	*C. glabrata*	*C. tropicalis*	*C. albicans*	*C. glabrata*	*C. tropicalis*	*C. albicans*	*C. glabrata*	*C. tropicalis*
Acids	3	0	0	0	2	3	2	2	3	3	3	3	3
Alcohols	15	15	15	15	15	15	15	15	15	15	15	15	15
Aldehydes	20	19	20	20	20	20	20	20	20	20	20	20	20
Hydrocarbons	17	17	17	17	17	17	17	16	16	16	16	16	16
Esters	16	13	14	13	15	16	14	16	16	15	16	15	15
Ketones	15	13	15	14	12	13	13	12	13	12	12	12	11
Monoterpenic compounds	16	15	15	14	15	15	13	15	14	14	15	14	14
Sesquiterpenic compounds	13	12	8	7	13	13	10	12	13	13	12	13	13
Norisoprenoid	1	1	1	1	1	1	1	1	1	1	1	1	1
Phenols	4	4	4	4	4	4	4	4	4	4	4	4	4
Sulphur compounds	6	6	6	6	6	6	6	6	6	6	6	6	6
**TOTAL**	**126**	**115**	**115**	**111**	**120**	**123**	**115**	**119**	**121**	**119**	**120**	**119**	**118**

Table S3 – Metabolites detected for each *Candida* species over the time-course experiment. This information was used to construct the Figure 5 (total number of shared and unique metabolites of each *Candida* species over the time-course experiment).

Metabolite	Growth times/metabolites detected											
	6h			12h			24h			48h		
	C. albicans	*C. glabrata*	*C. tropicalis*	*C. albicans*	*C. glabrata*	*C. tropicalis*	*C. albicans*	*C. glabrata*	*C. tropicalis*	*C. albicans*	*C. glabrata*	*C. tropicalis*
Octanoic acid	-	-	-	✓	✓	✓	✓	✓	✓	✓	✓	✓
Nonanoic acid	-	-	-	-	✓	-	-	✓	✓	✓	✓	✓
Decanoic acid	-	-	-	✓	✓	✓	✓	✓	✓	✓	✓	✓
1-Propanol	✓	✓	✓	✓	✓	✓	✓	✓	✓	✓	✓	✓
2-Methyl-1-propanol	✓	✓	✓	✓	✓	✓	✓	✓	✓	✓	✓	✓
1-Butanol	✓	✓	✓	✓	✓	✓	✓	✓	✓	✓	✓	✓
2/3-Methyl-1-butanol	✓	✓	✓	✓	✓	✓	✓	✓	✓	✓	✓	✓
1-Hexanol	✓	✓	✓	✓	✓	✓	✓	✓	✓	✓	✓	✓
1-Heptanol	✓	✓	✓	✓	✓	✓	✓	✓	✓	✓	✓	✓
1-Octen-3-ol	✓	✓	✓	✓	✓	✓	✓	✓	✓	✓	✓	✓
2-Ethyl-1-hexanol	✓	✓	✓	✓	✓	✓	✓	✓	✓	✓	✓	✓
1-Octanol	✓	✓	✓	✓	✓	✓	✓	✓	✓	✓	✓	✓
1-Nonanol	✓	✓	✓	✓	✓	✓	✓	✓	✓	✓	✓	✓
1-Decanol	✓	✓	✓	✓	✓	✓	✓	✓	✓	✓	✓	✓
2-Undecanol	✓	✓	✓	✓	✓	✓	✓	✓	✓	✓	✓	✓
1-Dodecanol	✓	✓	✓	✓	✓	✓	✓	✓	✓	✓	✓	✓
2-Phenyl-2-propanol	✓	✓	✓	✓	✓	✓	✓	✓	✓	✓	✓	✓
2-Phenylethanol	✓	✓	✓	✓	✓	✓	✓	✓	✓	✓	✓	✓
Acetaldehyde	✓	✓	✓	✓	✓	✓	✓	✓	✓	✓	✓	✓
2-Propenal	✓	✓	✓	✓	✓	✓	✓	✓	✓	✓	✓	✓
2-Methyl-propanal	✓	✓	✓	✓	✓	✓	✓	✓	✓	✓	✓	✓
3-Methylbutanal	✓	✓	✓	✓	✓	✓	✓	✓	✓	✓	✓	✓
2-Butenal	✓	✓	✓	✓	✓	✓	✓	✓	✓	✓	✓	✓
2-Methyl-butanal	✓	✓	✓	✓	✓	✓	✓	✓	✓	✓	✓	✓
3-Methyl-2-butenal	-	✓	✓	✓	✓	✓	✓	✓	✓	✓	✓	✓
Hexanal	✓	✓	✓	✓	✓	✓	✓	✓	✓	✓	✓	✓
Octanal	✓	✓	✓	✓	✓	✓	✓	✓	✓	✓	✓	✓
Nonanal	✓	✓	✓	✓	✓	✓	✓	✓	✓	✓	✓	✓
Decanal	✓	✓	✓	✓	✓	✓	✓	✓	✓	✓	✓	✓
2-Undecenal	✓	✓	✓	✓	✓	✓	✓	✓	✓	✓	✓	✓
Dodecanal	✓	✓	✓	✓	✓	✓	✓	✓	✓	✓	✓	✓
Benzaldehyde	✓	✓	✓	✓	✓	✓	✓	✓	✓	✓	✓	✓
Benzeneacetaldehyde	✓	✓	✓	✓	✓	✓	✓	✓	✓	✓	✓	✓
2,5-Dimethylbenzaldehyde	✓	✓	✓	✓	✓	✓	✓	✓	✓	✓	✓	✓

4-(1-Methylethyl)-benzaldehyde	✓	✓	✓	✓	✓	✓	✓	✓	✓	✓	✓	✓
α- Ethylidenbenzeneacetaldehyde	✓	✓	✓	✓	✓	✓	✓	✓	✓	✓	✓	✓
2,4,6-Trimethylbenzaldehyde	✓	✓	✓	✓	✓	✓	✓	✓	✓	✓	✓	✓
3,5-di-tert-Butyl-4-hydroxybenzaldehyde	✓	✓	✓	✓	✓	✓	✓	✓	✓	✓	✓	✓
Nonane	✓	✓	✓	✓	✓	✓	✓	✓	✓	✓	✓	✓
1-Dodecene	✓	✓	✓	✓	✓	✓	✓	✓	✓	✓	✓	✓
Heptadecane	✓	✓	✓	✓	✓	✓	✓	✓	✓	✓	✓	✓
Toluene	✓	✓	✓	✓	✓	✓	✓	✓	✓	✓	✓	✓
Ethylbenzene	✓	✓	✓	✓	-	✓	-	-	-	-	-	-
Styrene	✓	✓	✓	✓	✓	✓	✓	✓	✓	✓	✓	✓
Isopropylbenzene	✓	✓	✓	✓	✓	✓	✓	✓	✓	✓	✓	✓
Propylbenzene	✓	✓	✓	✓	✓	✓	✓	✓	✓	✓	✓	✓
α-Methylstyrene	✓	✓	✓	✓	✓	✓	✓	✓	✓	✓	✓	✓
1,2,4,5-Tetramethylbenzene	✓	✓	✓	✓	✓	✓	✓	✓	✓	✓	✓	✓
1,4-Di-tert-butylbenzene	✓	✓	✓	✓	✓	✓	✓	✓	✓	✓	✓	✓
Biphenyl	✓	✓	✓	✓	✓	✓	✓	✓	✓	✓	✓	✓
1-Butylheptylbenzene	✓	✓	✓	✓	✓	✓	✓	✓	✓	✓	✓	✓
1-Propyloctylbenzene	✓	✓	✓	✓	✓	✓	✓	✓	✓	✓	✓	✓
1-Butyloctylbenzene	✓	✓	✓	✓	✓	✓	✓	✓	✓	✓	✓	✓
1-Propylnonylbenzene	✓	✓	✓	✓	✓	✓	✓	✓	✓	✓	✓	✓
1-Ethyldecylbenzene	✓	✓	✓	✓	✓	✓	✓	✓	✓	✓	✓	✓
Ethyl acetate	✓	✓	✓	✓	✓	✓	✓	✓	✓	✓	✓	✓
Ethyl propanoate	-	✓	-	✓	✓	✓	✓	✓	✓	✓	✓	✓
Isobutyl acetate	✓	✓	✓	✓	✓	✓	✓	✓	✓	✓	✓	✓
Ethyl butanoate	✓	✓	✓	✓	✓	✓	✓	✓	✓	✓	✓	✓
Butyl ethanoate	✓	✓	✓	✓	✓	✓	✓	✓	✓	✓	✓	✓
Isoamyl ethanoate	-	-	✓	✓	✓	✓	✓	✓	✓	✓	✓	✓
Hexyl ethanoate	✓	✓	✓	✓	✓	✓	✓	✓	✓	✓	-	✓
Ethyl octanoate	✓	✓	✓	✓	✓	✓	✓	✓	✓	✓	✓	✓
Ethyl nonanoate	✓	✓	-	✓	✓		✓	✓	-	✓	✓	-
3-Hydroxy-2,4,4-trimethylpentyl 2-methypropanoate	✓	✓	✓	✓	✓	✓	✓	✓	✓	✓	✓	✓
Propanoic acid, 2-methyl-, 1-(1,1-dimethylethyl)--2-methyl-1,3-propanediyl ester	✓	✓	✓	✓	✓	✓	✓	✓	✓	✓	✓	✓
Lauryl acetate	✓	✓	✓	✓	✓	✓	✓	✓	✓	✓	✓	✓
Isopropyl myristate	✓	✓	✓	✓	✓	✓	✓	✓	✓	✓	✓	✓
Methyl benzoate	✓	✓	✓	✓	✓	✓	✓	✓	✓	✓	✓	✓
2-Benzylacrylic acid methyl ester	✓	✓	✓	✓	✓	✓	✓	✓	✓	✓	✓	✓
2-Octyl benzoate	-	-	-	-	✓	-	✓	✓	✓	✓	✓	✓

2-Propanone	✓	✓	✓	✓	✓	✓	✓	✓	✓	✓	✓	✓
2-Butanone	-	✓	✓	-	-	-	-	-	-	-	-	-
2-Pentanone	✓	✓	✓	✓	-	✓	✓	-	-	✓	-	-
2,3-Pentanedione	✓	✓	✓	-	✓	✓	-	✓	✓	-	-	-
4-Methyl-2-pentanone	✓	✓	✓	✓	✓	✓	✓	✓	✓	✓	✓	✓
3-Penten-2-one	-	✓	✓	-	✓	✓	-	✓	✓	-	✓	✓
2,3-Heptanedione	✓	✓	✓	✓	✓	✓	✓	✓	✓	✓	✓	✓
5-Methyl-2-hexanone	✓	✓	✓	✓	✓	✓	✓	✓	✓	✓	✓	✓
4-Heptanone	✓	✓	-	✓	✓	-	✓	✓	-	✓	✓	-
6-Methyl-2-heptanone	✓	✓	✓	✓	✓	✓	✓	✓	✓	✓	✓	✓
2-Nonanone	✓	✓	✓	✓	✓	✓	✓	✓	✓	✓	✓	✓
Phenylacetone	✓	✓	✓	✓	✓	✓	✓	✓	✓	✓	✓	✓
1-Phenyl-1-butanone	✓	✓	✓	✓	✓	✓	✓	✓	✓	✓	✓	✓
2-Undecanone	✓	✓	✓	✓	✓	✓	✓	✓	✓	✓	✓	✓
2-Tridecanone	✓	✓	✓	✓	✓	✓	✓	✓	✓	✓	✓	✓
α-Pinene	✓	✓	✓	✓	✓	✓	✓	✓	✓	✓	✓	✓
Verbenene	✓	✓	✓	-	-	-	-	-	-	-	-	-
3-Carene	✓	✓	✓	✓	✓	✓	✓	✓	✓	✓	✓	✓
Limonene	✓	✓	✓	✓	✓	✓	✓	✓	✓	✓	✓	✓
β-Ocimene	-	-	-	✓	✓	-	✓	✓	✓	✓	✓	✓
Dihydromyrcenol	✓	✓	✓	✓	✓	✓	✓	✓	✓	✓	✓	✓
Linalool tetrahydride	✓	✓	✓	✓	✓	✓	✓	✓	✓	✓	✓	✓
Linalool	✓	✓	✓	✓	✓	✓	✓	✓	✓	✓	✓	✓
Fenchyl alcohol	✓	✓	✓	✓	✓	✓	✓	✓	✓	✓	✓	✓
Dihydroterpineol	✓	✓	✓	✓	✓	✓	✓	✓	✓	✓	✓	✓
Pinocarvone	✓	✓	✓	✓	✓	✓	✓	✓	✓	✓	✓	✓
Borneol	✓	✓	✓	✓	✓	✓	✓	✓	✓	✓	✓	✓
Carvone	✓	✓	✓	✓	✓	✓	✓	✓	✓	✓	✓	✓
Geraniol	✓	✓		✓	✓	✓	✓	✓	✓	✓	✓	✓
Endobornyl acetate	✓	✓	✓	✓	✓	✓	✓	✓	✓	✓	✓	✓
β-Terpenyl acetate	✓	✓	-	✓	✓	-	✓	-	-	-	-	-
α-Copaene	✓	✓	✓	✓	✓	✓	✓	✓	✓	✓	✓	✓
Longifolene	✓	✓	✓	✓	✓	✓	✓	✓	✓	✓	✓	✓
β-Farnesene	✓	✓	✓	✓	✓	✓	✓	✓	✓	✓	✓	✓
Geranyl acetone	✓	✓	✓	✓	✓	✓	✓	✓	✓	✓	✓	✓
α-Curcumene	✓	✓	✓	✓	✓	✓	✓	✓	✓	✓	✓	✓
α-Farnesene isomer	✓	-	-	✓	✓	✓	✓	✓	✓	✓	✓	✓
α-Farnesene isomer	✓	-	✓	✓	✓	✓	✓	✓	✓	✓	✓	✓
Calamenene	✓	✓	✓	✓	✓		✓	✓	✓	✓	✓	✓
Nerolidol	✓	✓	-	✓	✓	✓	✓	✓	✓	✓	✓	✓
Caryophyllene oxide	-	✓	-	✓	✓	✓	-	✓	✓	-	✓	✓

2,3-Dihydrofarnesol	✓	-	-	✓	✓	✓	✓	✓	✓	✓	✓	✓
E,E-Farnesol	✓	-	-	✓	✓	-	✓	✓	✓	✓	✓	✓
Farnesal	✓	-	-	✓	✓	-	✓	✓	✓	✓	✓	✓
α -Methylionone	✓	✓	✓	✓	✓	✓	✓	✓	✓	✓	✓	✓
2-(1,1-Dimethylethyl)-4-methylphenol	✓	✓	✓	✓	✓	✓	✓	✓	✓	✓	✓	✓
5-Methyl-2,4-diisopropylphenol	✓	✓	✓	✓	✓	✓	✓	✓	✓	✓	✓	✓
2,4-Bis(1,1'-dimethylethyl)phenol	✓	✓	✓	✓	✓	✓	✓	✓	✓	✓	✓	✓
Nonylphenol	✓	✓	✓	✓	✓	✓	✓	✓	✓	✓	✓	✓
Methanethiol	✓	✓	✓	✓	✓	✓	✓	✓	✓	✓	✓	✓
Dimethyl disulfide	✓	✓	✓	✓	✓	✓	✓	✓	✓	✓	✓	✓
Thiazole	✓	✓	✓	✓	✓	✓	✓	✓	✓	✓	✓	✓
2-Methyl-thiophene	✓	✓	✓	✓	✓	✓	✓	✓	✓	✓	✓	✓
3-(Methylthio)propanal	✓	✓	✓	✓	✓	✓	✓	✓	✓	✓	✓	✓
Dimethyl trisulfide	✓	✓	✓	✓	✓	✓	✓	✓	✓	✓	✓	✓

✓ Presence of metabolite

- Absence of the metabolite
